# The disproportionate importance of long‐unburned forests and woodlands for reptiles

**DOI:** 10.1002/ece3.4561

**Published:** 2018-10-17

**Authors:** Kelly M. Dixon, Geoffrey J. Cary, Graeme L. Worboys, Philip Gibbons

**Affiliations:** ^1^ Fenner School of Environment and Society The Australian National University Acton ACT Australia

**Keywords:** biodiversity, fire management, fire mosaic, prescribed burning, time since fire

## Abstract

Our understanding of the impacts of time since fire on reptiles remains limited, partly because there are relatively few locations where long‐term, spatially explicit fire histories are available. Such information is important given the large proportion of some landscapes that are managed with frequent prescribed fire to meet fuel management objectives. We conducted a space‐for‐time study across a landscape in southeastern Australia where the known fire history spanned 6 months to at least 96 years. Four methods were used to survey reptiles in 81 forest and woodland sites to investigate how time since fire (TSF), habitat, and environmental variables affect reptile richness, abundance, and composition. We used generalized linear models, generalized linear mixed‐effects models, PERMANOVA, and SIMPER to identify relationships between the reptile assemblage (richness, abundance, and composition, respectively) and TSF, habitat, and environmental variables. All three reptile metrics were associated with TSF. Reptile richness and abundance were significantly higher in sites >96 years postfire than younger fire ages (0.5–12 years). Reptile composition at long‐unburned sites was dissimilar to sites burned more recently but was similar between sites burned 0.5–2 and 6–12 years prior to sampling. *Synthesis and applications.* Long‐unburned forests and woodlands were disproportionately more important for reptile richness and abundance than areas burned 6 months to 12 years prior to sampling. This is important given that long‐unburned areas represent <8% of our study area. Our results therefore suggest that reptiles would benefit from protecting remaining long‐unburned areas from fire and transitioning a greater proportion of the study area to long‐unburned. However, some compositional differences between the long‐unburned sites and sites 0.5–12 years postfire indicate that maintaining a diversity in fire ages is important for conserving reptile diversity.

## 1 INTRODUCTION

Prescribed burning is the most widely used management tool for reducing fuel in fire‐prone regions around the world (Fernandes & Botelho, 2003) and is increasingly applied for ecological purposes (Penman et al., 2011). However, the frequency that prescribed burning is required for fuel hazard reduction may not be consistent with biodiversity conservation objectives (Morrison, Buckney, Bewick, & Cary, 1996; Penman et al., 2011). While appropriate fire regimes for flora are relatively well known (Bradstock & Cohn, 2002), those for many faunal species remain poorly understood (Driscoll et al., 2010), resulting in many fire management plans omitting fauna altogether (Clarke, 2008). Reptiles are one taxonomic group that appear to be affected where a landscape shifts toward being dominated by shorter fire intervals (Abom & Schwarzkopf, 2016; Elzer et al., 2013; Nimmo et al., 2013; Valentine, Reaveley, Johnson, Fisher, & Wilson, 2012); however, knowledge of reptile responses to fire remains limited.

Reptile community succession after fire is complex and dependent not only upon changes in habitat structure and complexity (Letnic, Dickman, Tischler, Tamayo, & Beh, 2004; Smith, Bull, & Driscoll, 2013) but also factors such as trophic interactions, species movement, physiology (Smith, 2018), and behavioral adaptations (Pausas & Parr, 2018). Thus, it stands to reason that as habitat and interactions change over time, reptile community composition should respond in some predictable sequence when preferred resources become available (the “habitat‐accommodation” model of succession (Fox, 1982; Caughley, 1985)). Dominant pioneer and early seral‐stage reptile species generally utilize habitat features largely unaffected by fire, such as burrows (Smith et al., 2013) or rocks (Santos, Badiane, & Matos, 2016), and often feed on invertebrate species whose abundance is unchanged or increases immediately postfire (Griffiths & Christian, 1996; Letnic et al., 2004; Nicholson, Lill, & Andersen, 2006; Smith, 2018). Additionally, increased sunlight penetration and bare ground occurring after fire provide thermoregulatory opportunities and have been related to higher reptile abundance than in areas with greater cover and structure (Greenberg & Waldrop, 2008; Matthews, Moorman, Greenberg, & Waldrop, 2010; Moseley, Castleberry, & Schweitzer, 2003; Pinto, Bombi, & Bologna, 2006).

Shrubs and understorey may re‐establish as soon as 2 years after fire in some areas (Morrison et al., 1996) and remain at high density for at least four to five decades (Haslem et al., 2011). During this typically shrubbier seral stage, reptile species that require open areas may decline (Ballinger & Watts, 1995; Pinto et al., 2006), sometimes to the point of localized extinction (Jaggi & Baur, 1999). Additionally, dense understorey may inhibit dispersal of less vagile species, within even very short distances, thereby disrupting gene flow (Templeton, Robertson, Brisson, & Strasburg, 2001). However, reptile species that benefit from more structurally complex habitat begin to re‐occupy these areas (Caughley, 1985) and increase in abundance with increased structural complexity (Letnic et al., 2004; Pinto et al., 2006). Late succession species often rely on habitat attributes such as leaf litter (Smith et al., 2013) or coarse woody debris (CWD) (Croft, Hunter, & Reid, 2016; Haslem et al., 2011) that generally take time to re‐accumulate or develop after fire. In certain vegetation types, forests free of fire for many decades self‐thin, and shrubby understories senesce, this being characterized by a more open state (Kitzberger, Aráoz, Gowda, Mermoz, & Morales, 2012). Increased canopy and understorey gaps that provide higher insolation than surrounding areas may positively influence heliothermic reptile diversity (Brown & Nelson, 1993; Greenberg, 2001).

Despite some reptile species typically displaying distinct successional trends in some regions, reptile responses to TSF may vary across their geographic range and with climatic and environmental conditions (Nicholson et al., 2006; Nimmo, Kelly, Farnsworth, Watson, & Bennett, 2014), including not exhibiting an apparent response to TSF or following an ordered succession (Driscoll & Henderson, 2008; Farnsworth, Nimmo, Kelly, Bennett, & Clarke, 2014; Lindenmayer et al., 2008; Smith et al., 2013). Additionally, species presumed to be specialists of a particular seral stage may not be present until later in the successional stage if they also rely on specific resources that take time to develop. For example, a burrowing Australian agamid, *Ctenophorus cristatus*, was unexpectedly less abundant in recently burned than long‐unburned sites, likely due to lower amounts of CWD soon after fire (Driscoll & Henderson, 2008). Given the considerable variation in reptile responses, or lack thereof, to TSF and habitat complexity, determining beneficial landscape fire mosaics for reptiles is challenging. Nonetheless, a body of research has identified the disproportionate importance of long‐unburned areas, compared with those managed under shorter fire intervals, for vertebrates, including reptiles, and important habitat attributes (Croft et al., 2016; Giljohann, McCarthy, Kelly, & Regan, 2015; Haslem et al., 2012; Kelly et al., 2012; Kelly, Bennett, Clarke, & McCarthy, 2015; Taylor et al., 2012). Long‐unburned forested landscapes are becoming relatively rare in wildfire‐prone regions of the world, and where they exist often comprise a small proportion of the larger landscape.

In this study, we investigated reptile populations in *Eucalyptus* forests and woodlands spanning 6 months to at least 96 years since fire and experiencing zero to six prescribed and wildfires in this period. At the time of sampling, the majority of our study area had a TSF of ≤12 years and <10% was long‐unburned (>96 years since fire). The primary objective of this study was to determine how reptile richness and abundance vary in recently burned to very long‐unburned forests and woodlands, to ascertain the differences in reptile assemblages across the seral stages present, and to identify habitat and environmental variables that may influence reptile communities.

## 2 MATERIALS AND METHODS

### 2.1 Study area

We conducted our study in Namadgi National Park (35°39′02.49″S, 148°50′46.79″E), an area that covers 106,095 ha (46%) of the Australian Capital Territory (ACT), southeastern Australia, and forms part of the Australian Alps network of national parks. Elevation across the study sites ranges from 738 to 1,651 m, and average annual rainfall varies from 616 to 1,426 mm. Coldest and warmest mean monthly temperatures range from −3.51 to 0.26°C and 20.19 to 27.32°C, respectively, based on predictions from ANUCLIM 6.1.1 (Xu & Hutchinson, 2011). Dominant vegetation classes in our study sites are dry sclerophyll forest, subalpine woodland, wet sclerophyll forest, and montane wet sclerophyll forest (Keith, 2004).

### 2.2 Fire history

We obtained a 96‐year fire history for our study area spanning 1920 to 2015 from the ACT Environment, Planning, and Sustainable Development Directorate. Of our 81 sites, 54 have experienced between one and six fires since 1920 and 27 have no record of fire. TSF across our study area ranged from 6 months to at least 96 years at the time of sampling (Supporting Information Figure S1). In 2003, an extensive wildfire burned at varying levels of severity across 91% of Namadgi National Park. Of the 53 sites that burned in 2003, 22 were treated with prescribed burning between 2009 and 2015, and 31 have not experienced fire since 2003. The level of severity (1–5) of the 2003 wildfire appeared to have no impact on vegetation density and structure at the time of sampling in these 31 sites that burned in 2003 but not subsequently (Dixon, Cary, Worboys, Seddon, & Gibbons, 2018). We recorded TSF as a categorical variable with three levels: 0.5–2 years (*n = *19), 6–12 years (*n = *35), and>96 years (*n = *27) due to low numbers of sites in some age classes, due to the large age gap between the longest unburned sites and all other ages, and because we could not determine precise TSF for sites that were fire‐free for at least 96 years.

### 2.3 Site selection

We employed a space‐for‐time approach to investigate reptile species richness, abundance, and composition as a function of TSF, habitat resources, and other environmental variables. We selected 81 forest and woodland sites (50 m × 20 m) from a random sample across nine strata derived from fire history and a topographic wetness index (TWI). TWI is calculated as log[specific catchment/slope] (Moore, Grayson, & Ladson, 1991) and is a relative measure of available soil moisture based on topography and drainage (Kopecký & Čížková, 2010). TWI can affect the number of strata and total biomass and therefore structure of forest (Specht & Specht, 1999). Reptile composition often changes with vegetation structure (Letnic et al., 2004; Smith et al., 2013); therefore, TWI may be associated with reptile richness, abundance, and community composition. We grouped fire frequency (number of fires on record) into three classes: 0 fires, 1–3 fires, and 4–6 fires. TWI was grouped into three classes for site stratification which were derived from a 20 m resolution digital elevation model: −5.1 to −2, −1.99 to 0, and 0.01 to 2.5. Values above 2.5 represented standing water and were therefore omitted. We randomly selected nine sites in each of the nine strata (*n = *81) and ensured a minimum distance between sites of 500 m to minimize spatial autocorrelation.

### 2.4 Response variables

We used three metrics to determine reptile community response to fire: species richness, abundance, and composition. We conducted reptile surveys over 2 years (year 1: 2015–2016 and year 2: 2016–2017) between October and January, the Austral spring and summer, when reptiles are most active. We calculated species richness as the number of species and abundance as the total number of individuals of each species recorded at each site by all survey methods during each survey period. A combination of survey methods is often required to enable representation of reptile assemblages (Michael, Cunningham, Donnelly, & Lindenmayer, 2012). We therefore surveyed reptiles using four methods: active searches, visual searches, substrate searches, and camera trapping. All reptile searches were performed by KMD and one other person who was trained in reptile observational surveys and familiar with the identification of reptiles in our study area. Reptiles were identified to species level where possible using Wilson and Swan (2013) and Cogger (2014) and named using the taxonomic nomenclature in Cogger (2014).

We conducted two visual (*n = *162) and two active (*n = *162) time‐ and‐area‐constrained searches (20 min × 0.1 ha^−1^) (MacNally & Brown, 2001; Michael et al., 2012) at each site over the study period. One visual survey and one active survey were undertaken each year. Visual searches aimed at basking reptiles were conducted in bright sunshine within a temperature range of 16–30°C. Active searches aimed at basking and sheltering or cryptic reptiles were conducted in sunny or cloudy weather within a temperature range of 14–30°C. Both search types involved the two observers starting 10 m apart and walking slowly forward in a zigzag recording all reptiles seen within a 180° arc (MacNally & Brown, 2001) taking 20 min to walk the length and width of the 50 × 20 m site. Active searches were conducted similar to visual searches, but also involved prising loose bark from trees, turning logs and rocks, and raking through leaf litter.

At each site, we set out an artificial refuge station consisting of four concrete roofing tiles and a stack of two pieces of corrugated iron cut to approximately 1 m^2^ (Michael et al., 2012; Reading, 1997). We performed two checks each year of the artificial refuge (“substrate search”): one in spring and one in summer (*n = *324). We recorded all reptile species found on, under, or between, the substrate.

One Reconyx HC550 HyperFire camera trap was employed at each site for a period of 2 weeks in each survey period (*n* = 2,268 nights). Cameras were set at a vertical overhead orientation mounted on a star picket at a height of 70 cm between the ground and the face of the camera to enable identification of small skink species (Welbourne, 2013). The camera's field of view (~ 60 × 45 cm) was cleared of vegetation where we pegged a 30 × 30 cm cork tile baited with 10 ml of sesame oil (Diete, Meek, Dixon, Dickman, & Leung, 2016) and 10 ml of a mixture of sardine oil and rice bran oil. Cork allows a temperature difference between the ground and a reptile to enable the animal to trigger the camera (Welbourne, 2013). Cameras were set to fast shutter, capturing three images per trigger with no delay between triggers and we replenished bait oils after 1 week.

### 2.5 Potential explanatory variables

We measured eight habitat variables and recorded nine environmental and fire variables at each site that are likely to influence reptiles. Eight noncorrelated explanatory variables were used in the regression models (Table 1). Litter, ground vegetation, and shrub measurements were taken from five measurements in a 1 m radius (plot) for litter and a 2 m radius (plot) for ground stratum and shrub cover. The average of 10 plots per 50‐m transect was used as the final measurement. A description of variables excluded from models due to correlation with other variables is provided in Supporting Information Table S1.

**Table 1:** Summary of noncorrelated explanatory variables used in regression models

Variable	Description
Forest type	Vegetation class (Keith, 2004) divided into three classes: Dry sclerophyll forest, subalpine woodland, wet and montane wet sclerophyll forest
Aspect	Aspect of each site as one of four categorical factors (north, east, south, and west)
CWD m^3^/ha	Volume (m^3^) of CWD at each site. We measured the diameter and length of every piece of CWD ≥10 cm in diameter and ≥100 cm in length in a 20 × 20 m fixed area plot (Woldendorp, Keenan, & Ryan, 2002) in each site
Litter cover %	The average percentage of surface bark and litter cover
Ground cover %	The average horizontal percentage cover and height of grass and near‐surface vegetation (<50 cm high)
Shrub cover %	The average percentage cover of shrubs and vegetation (>50–300 cm high)
Rock cover %	The percentage of rock cover along each transect (50 m). Point intersect sampling was used by running a 50‐m transect through each site and measuring every rock >10 cm that touched the tape. The total length of all rocks was divided by the transect length

### 2.6 Statistical analyses

We used generalized linear models (GLMs) and generalized linear mixed‐effects models (GLMMs) and an information theoretic approach (Burnham & Anderson, 2002) to examine the influence of TSF, habitat, and environmental variables (explanatory variables) on reptile richness and abundance (response variables). We tested for spatial autocorrelation of the residuals of the best‐fitting model using Moran's *I*. Prior to fitting the regression models, we tested continuous explanatory variables for multicollinearity using Pearson's correlation coefficient and eliminated one of each pair of highly correlated variables (*r* ≥ 0.7). There was evidence of some correlation between TSF and CWD (*r = *0.63) and TSF and shrub cover proportion (*r = *0.61), but we included these potential explanatory variables in models. We log‐transformed values for CWD volume because untransformed values were highly skewed. Analyses were conducted in the R statistical package (R Core Development Team, 2013).

We conducted likelihood‐ratio tests and compared the Poisson and negative binomial models to determine the appropriate model family for response variables. Abundance models displayed over‐dispersion while richness did not. Thus, negative binomial regression was used for models of abundance and Poisson regression was used for models of richness. We compared models using Akaike's information criterion corrected for small samples (AIC_c_). We ranked models using AIC_c_ weights (AIC_c_ *W*_i_), which can be interpreted as the probability that the model is the best among the set of candidate models. Models with the lowest AIC_c_ value and Δ AIC_c_ ≤2 relative to the lowest AIC_c_ value have substantial support (Burnham & Anderson, 2002). For the GLMM fit to predict reptile richness, the variance attributed to the random effect of site was zero and the effect of survey period was not significant (*p* = 0.287). Therefore, we pooled richness data for both survey periods and used GLMs to predict this response variable. Model fitting was undertaken using the GlmmTMB package, and model selection was undertaken using the MuMIn package (Barton, 2018). We illustrated predictions (plots) for individual terms from our GLMs following the typical convention for term plots in most statistical software (including R): The effect of each variable is predicted while fixing all covariates at their mean (if the covariate is continuous) or at the level of a factor with the highest sample size (if the covariate is a factor).

To identify variables associated with dissimilarities in reptile composition between sites, we used permutational multivariate analysis of variance (PERMANOVA) (Anderson, 2001) using the adonis function in the vegan package within R (Oksanen et al., 2018). We used a matrix of sites by species populated with the total abundance for each species pooled across the 2 years of observation. These data were standardized using the Wisconsin double standardization, and we used the Bray–Curtis measure of dissimilarity. We initially fitted a model containing all potential explanatory variables and sequentially removed variables where *p* > 0.05. To identify the relative contribution of individual species to dissimilarities between levels of TSF, we used the SIMPER function, also within the vegan package in R (Oksanen et al., 2018). To illustrate differences in reptile composition between levels of TSF, we calculated the average distance to the centroid for each level of TSF in multivariate space using the betadisper function in the vegan package (Oksanen et al., 2018) and reduced these distances to principal coordinates.

## 3 RESULTS

In total, we recorded 3,216 individuals from 21 reptile species in four families at 79 of the 81 sites (Table 2). We combined *Eulamprus heatwolei* and *E. tympanum*, which appear superficially very similar, as “*Eulamprus* spp.” for analyses, making the total reptile richness 20. We were unable to identify 66 skinks to species level; thus, we excluded these from analyses. Observations were dominated by the generalist skinks *Pseudemoia entrecasteuaxii* (49% of observations) and *Lampropholis guichenoti* (24% of observations), though there were seven species for which there were more than 40 observations (Table 2). There was not one survey method alone that recorded all 20 species (Table 2, Supporting Information, Table S7, Figure S2).

**Table 2:** Number of individuals of reptile species observed during this study in the different time since fire categories by survey method

Family	Common name	Species name	Time since fire (years)			Survey method				Total
			0.5–2	6–12	>96	Act	Vis	Cam	Sub	
Agamidae	Jacky lizard	*Amphibolurus muricatus*	0	7	16	8	9	6	0	23
	Mountain heath dragon	*Rankinia diemensis*	1	3	1	0	3	2	0	5
Elapidae	Highland copperhead	*Austrelaps ramsayi*	1	2	5	1	4	0	3	8
	White‐lipped snake	*Drysdalia coronoides*	4	2	12	0	3	0	15	18
	Red‐bellied black snake	*Pseudechis porphyriacus*	0	1	1	2	0	0	0	2
	Eastern brown snake	*Pseudonaja textilis*	4	1	5	2	4	4	0	10
Scincidae	Bold‐striped skink	*Acritoscincus duperreyi*	4	5	23	12	7	3	10	32
	Red‐throated skink	*Acritoscincus platynotum*	5	4	21	11	6	9	4	30
	Mccoy's skink	*Anepischetosia maccoyi*	9	19	30	3	0	0	55	58
	Cunningham's skink	*Egernia cunninghami*	0	0	8	1	2	5	0	8
	Black rock skink	*Egernia saxatilis intermedia*	4	1	8	9	4	0	0	13
	Water skink	*Eulamprus* spp.[Link]	28	24	26	23	33	21	1	78
	Southern earless skink	*Hemiergis talbingoensis talbingoensis*	2	2	10	1	0	0	13	14
	Garden sun skink	*Lampropholis delicata*	0	1	55	18	24	4	10	56
	Grass sun skink	*Lampropholis guichenoti*	82	170	521	339	303	65	66	773
	Sun skink species	*Lampropholis* sp.[Link]	0	3	4	0	0	7	0	7
	White's skink	*Liopholis whitii*	4	4	35	19	17	6	1	43
	Southern grass skink	*Pseudemoia entrecasteauxii*	63	179	1,340	743	715	60	64	1582
	Spencer's skink	*Pseudemoia spenceri*	13	8	340	185	176	0	0	361
	Grass skink species	*Pseudemoia* sp.[Link]	0	0	2	0	0	1	1	2
	Blotched blue‐tongue lizard	*Tiliqua nigrolutea*	6	13	12	0	7	24	0	31
Varanidae	Rosenberg's monitor	*Varanus rosenbergi*	0	2	3	0	2	3	0	5

Notes

Act: active searches; Cam: camera trapping; Sub: artificial substrate searches; Vis: visual searches.

Two morphologically similar species of water skink (*Eualmprus heatwolei* and *E. tympanum*) were difficult to distinguish and are combined here for analyses.

Individuals of these genera were unable to be distinguished to species level and are presented here for information, however, were not included in analyses.

### 3.1 Reptile richness and abundance

**Figure 1:**
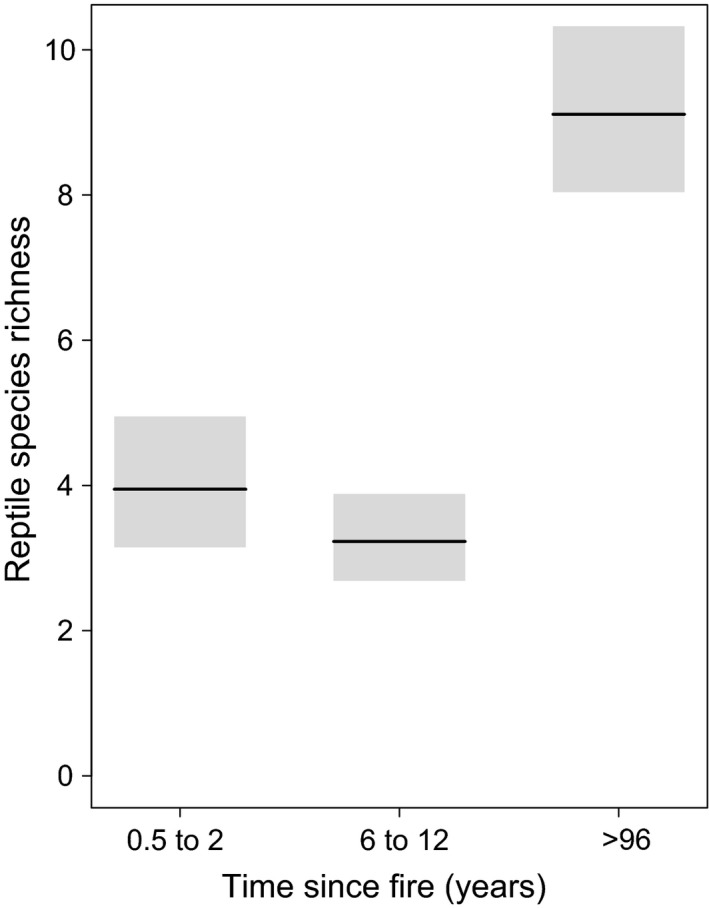
Predicted reptile richness (mean ± 95% confidence interval) with changes in time since last fire. Predictions are based off model 1.1 (Table 3)

Time since fire was the only explanatory variable in the best‐fitting model (model 1.1, Table 3) for predicting reptile richness. However, high‐ranking models (Δ AIC_c_ ≤2 relative to the best model) included TSF and one or two habitat variables (models 1.2–1.5, Table 3). Long‐unburned sites (>96 years) supported significantly higher species richness than sites 0.5–2 or 6–12 years postfire (Figure 1). While sites burned within 0.5–2 years supported slightly more species than those burned 6–12 years previously, there was no significant difference between these fire ages (*p* = 0.177) (Supporting Information, Table S2).

**Table 3:** Top models for reptile richness and abundance

Model	*df*	Log‐likelihood	AIC_c_	ΔAIC_c_	AIC_c_ W_i_
Reptile richness					
1.1 TSF	3	−160.380	327.1	0.00	0.229
1.2 TSF + rock cover + ground cover	5	−158.412	327.6	0.55	0.174
1.3 TSF + ground cover	4	−159.674	327.9	0.80	0.153
1.4 TSF + CWD	4	−160.152	328.8	1.76	0.095
1.5 TSF + litter cover	4	−160.320	329.2	2.10	0.080
1.6 TSF + shrub cover	4	−160.363	329.3	2.18	0.077
1.7 TSF + CWD + rock cover	5	−159.382	329.6	2.49	0.066
1.8 TSF + CWD + ground cover	5	−159.429	329.7	2.59	0.063
Reptile abundance					
2.1 TSF + CWD + ground cover + year	8	−544.618	1,106.2	0.00	0.301
2.3 TSF + CWD + shrub cover + ground cover + year	9	−543.990	1,107.2	0.99	0.184
2.2 TSF + CWD + litter cover + ground cover + shrub cover + year	10	−543.192	1,107.8	1.66	0.131
2.4 TSF + CWD + year	7	−546.872	1,108.5	2.29	0.096
2.6 TSF + ground cover + year	7	−547.016	1,108.8	2.58	0.083
2.5 TSF + CWD + shrub cover + year	8	−546.181	1,109.3	3.12	0.063

Models with Δ AIC_c_ <4 relative to the lowest AIC_c_ value are shown. Models for abundance included a fixed effect of year and a random effect of site. Coefficients and standard errors for the best‐fitting models and the complete list of models are presented in Supporting Information.

CWD: coarse woody debris; TSF: time since fire.

**Figure 2:**
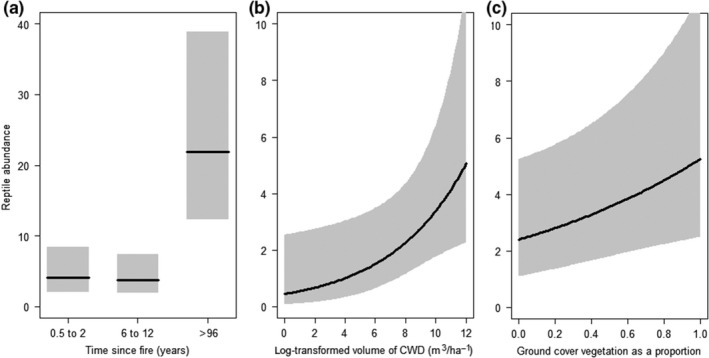
Predicted reptile abundance (mean ± 95% confidence interval) with changes in (a) time since fire, (b) log‐transformed volume of coarse woody debris (CWD) (m^3^ ha^−1^), and (c) ground cover vegetation as a proportion. Predictions are based off model 2.1 (Table 3)

The variables TSF, ground cover proportion, and CWD volume were included in the best‐fitting model (model 2.1, Table 3) for predicting reptile abundance (Figure 2a–c). Additionally, survey year affected reptile abundance, which was slightly higher in 2016 than in 2015.

### 3.2 Reptile composition

**Figure 3:**
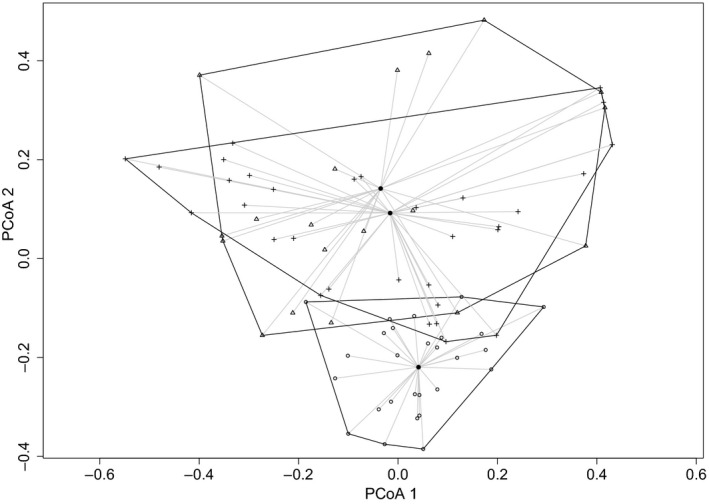
Dissimilarity between reptile assemblages on sites grouped by time since fire. Triangles = sites 0.5–2 years since fire, crosses = sites 6–12 years since fire, and open circles = sites >96 years since fire. The centroid for each level of times since fire is illustrated by the solid circles.

Our analysis using PERMANOVA indicated that dissimilarities in reptile composition between sites were most strongly associated with TSF (*F* = 4.87, *df*_2,78_, *p* = 0.001) and, to a lesser extent, elevation (*F* = 3.67, *df*_1,78_, *p = *0.001) and CWD (*F* = 2.16, *df*_1,78_, *p = *0.016). Reptile composition appeared to be relatively similar between the two younger fire age categories (0.5–2 and 6–12 years postfire), with greater dissimilarity to long‐unburned sites (Figure 3). Two species, *Pseudemoia entrecasteauxii* and *L. guichenoti,* were influential in the compositional differences between all fire ages (Table 4). We recorded 63 and 82 individuals of these species respectively at sites 0.5–2 years postfire, 179 and 170 individuals at sites 6–12 years postfire and 1,340 and 521 individuals on sites >96 years postfire. A third species, *P. spenceri,* was influential in compositional differences between the recently burned sites and long‐unburned sites. We recorded 13 individuals on sites 0.5–2 years postfire, eight individuals on sites 6–12 years postfire, and 340 individuals on sites >96 years postfire. *Eulamprus* spp. was the most important contributor in compositional differences between sites 0.5–2 and 6–12 years postfire, although these differences were not strong. The remaining 16 species all made minor contributions to the differences in assemblages across the different fire ages (Supporting Information Tables S6–S8).

**Table 4:** Results from the SIMPER analysis of dissimilarity showing the three main reptile species that contribute the most variance between the three fire age categories

Species	Average abundance			Average dissimilarity			% contribution			Cumulative contribution (%)		
				Contrast			Contrast			Contrast		
	0.5–2	6–12	>96	A	B	C	A	B	C	A	B	C
*Pseudemoia entrecasteauxii*	3.58	5.48	49.37	0.392	0.182	0.374	50.06	29.51	49.61	50.06	29.51	49.61
*Lampropholis guichenoti*	5.00	5.24	18.70	0.170	0.187	0.167	21.70	30.23	22.20	71.76	59.74	71.81
*Pseudemoia spenceri*	0.68	0.24	12.59	0.089	–	0.088	11.42	–	11.70	83.18	–	83.51
*Eulamprus* spp.	1.42	0.76	0.96	–	0.067	–	–	10.93	–	–	70.67	–

Contrasts of fire age categories are between: A = 0.5–2 years and >96 years; B = 0.5–2 years and 6–12 years; and C = 6–12 years and >96 years. Average dissimilarity and % contribution values are shown only for the three species that make the greatest contribution to dissimilarity in each contrast. The results for all species can be found in Supporting Information Table S6.

## 4 DISCUSSION

We sought to determine whether TSF affects reptile richness, abundance, and community composition and identify which habitat features contribute to any observed differences. Our results indicated that long‐unburned forests and woodlands are disproportionately important for reptiles. Richness and abundance were highest in forests and woodlands that have been fire‐free for at least 96 years. There was a significant difference in the composition of reptile communities between sites with different fire ages. Greatest differences in reptile composition differed between the long‐unburned sites (>96 years since fire) and the younger fire ages (0.5–2 and 6–12 years since fire). CWD and the percentage cover of vegetation at ground level were habitat variables most strongly associated with reptile abundance.

There were no sites in our study aged between 13 and >96 years postfire, and therefore, we were unable to determine the nature of reptile assemblages within this period. It is possible that reptile assemblage on sites between 13 and 96 years postfire would be different to those observed in our study (Smith et al., 2013). Forest understorey begins to senesce approximately 20–40 years postfire (Haslem et al., 2011; Taylor, McCarthy, & Lindenmayer, 2014). In mallee shrubland, intermediate times since fire (11–35 years) provide habitat that is more beneficial for fire‐sensitive reptiles than habitat <11 years or >35 years postfire (Nimmo et al., 2013). It is plausible that in the fire ages absent from our study, shrub and understorey cover would be higher than in our long‐unburned sites, but lower than our younger postfire aged sites, and vice versa for CWD (Haslem et al., 2011; Vesk, Nolan, Thomson, Dorrough, & Nally, 2008). These habitat changes may offer different shelter, thermoregulatory, and dietary resources to those available in our study area at the time of sampling, and thus result in a different reptile community composition. Variables not measured in this study, such as canopy cover and solar radiation, may have some influence on reptile assemblage along with time since fire (Pike, Webb, & Shine, 2010). However, canopy cover varies with forest type and vegetation type was not an important predictor variable in any of our models with Δ AIC_c_ < 5.

### 4.1 Richness and abundance

Time since fire was a strong predictor of reptile richness and abundance (Table 3, Figures 1 and 2). However, key faunal resources are strongly affected by TSF (Croft et al., 2016; Haslem et al., 2011), and therefore, TSF may be a surrogate for several habitat variables. Indeed, CWD was a variable that appeared in high‐ranking models (ΔAIC_c_ < 2) we selected for predicting the richness and abundance of reptiles (Table 3, Figure 2). Our results are consistent with previous research that reptile abundance significantly increases with higher volumes of CWD (Figure 2b) (Manning, Cunningham, & Lindenmayer, 2013; Shoo, Wilson, Williams, & Catterall, 2014). CWD provides shelter, basking substrate, egg deposition sites, and improved thermal conditions for reptiles, and habitat for invertebrates upon which reptiles feed (Harmon et al., 1986). CWD accumulates slowly and may take up to a century to reach levels sufficient for benefitting biodiversity (Manning, Lindenmayer, & Cunningham, 2007; Vesk et al., 2008), but the volume and suitability of CWD are diminished with frequent fire (Croft et al., 2016; Haslem et al., 2011). In our study area, long‐unburned sites supported significantly higher levels of CWD than recently burned sites (K. M. Dixon, G. J. Cary, G. L. Worboys, P. Gibbons, unpublished data).

The cover of vegetation in the ground stratum (<0.5 m tall) was also a useful variable for predicting the abundance of reptiles (Table 3, Figure 2). Reduced cover of vegetation in the ground stratum is associated with lower reptile abundance in northern grasslands and woodlands within the ACT (Howland et al., 2014). Similarly, we predicted that reptile abundance was positively associated with the cover of vegetation in the ground stratum, albeit with a wide confidence interval (Figure 2c). However, not all reptile species benefit from increased cover of vegetation in the ground stratum and our results reflect responses by the most abundant species. For example, the abundance of a species from the family Agamidae (*Amphibolurus muricatus*) that was present in low numbers in our study declined with increasing grass cover in coastal southeastern Australia (Lindenmayer et al., 2008). Additionally, predatory species such as monitors or the frillneck lizard (*Chlamydosaurus kingii*) benefit from fire induced vegetation removal creating greater foraging opportunities (Griffiths & Christian, 1996). Vegetation type has been found by some to be more important for reptile richness and abundance than TSF or habitat structure (Lindenmayer et al., 2008; Santos et al., 2016; Valentine et al., 2012). However, in our study, forest type was not an important variable in any of the models with ΔAIC_c_ ≤5, presumably because our comparison was limited to forests and woodlands rather than vegetation types with greater structural contrasts, such as grasslands.

### 4.2 Community composition

Compositional differences in reptile assemblages in our study were most evident between long‐unburned sites and sites in the two younger postfire categories (Figure 3). We expected greater differences in composition between sites 0.5–2 and 6–12 years postfire due to a change from pioneer species to those that establish in the subsequent seral stage (Driscoll & Henderson, 2008; Smith et al., 2013). Regrowth of shrubs may sometimes occur so swiftly that species expected in the early seral state may not get the opportunity to establish (Lindenmayer et al., 2008). In our study sites, shrubs and understorey were often a similar height and cover in sites burned within 2 years to those burned 6–12 years prior to sampling (Dixon et al., 2018). Shrub cover was not included in the best model for predicting reptile abundance; however, it was an important explanatory variable in high‐ranking models (ΔAIC_c_ ≤ 2) (Table 3).

While our study did not specifically investigate reptile successional response, we found clear relationships between richness and abundance and time since fire, which may indicate some weak successional organization. The differences between recently burned and long‐unburned sites imply the reptile assemblage is composed of at least some fire‐sensitive species (Abom & Schwarzkopf, 2016).

### 4.3 Detection

Many studies investigating reptiles use a limited number of survey methods. Our study included four methods, which provided a broader sampling of the reptile community given not one of the methods detected all species. Seven species (35%) were detected using all survey methods (Supporting Information Figure S2, Table S9); however, detection rate was often uneven across method types. For example, all four methods detected *Eulamprus* spp., though only one individual was recorded under substrate while 98.7% of detections were split reasonably evenly between active searches, visual searches, and camera trapping. The leaf litter and burrowing skinks *Anepischetosia maccoyi* and *Hemiergis talbingoensis talbingoensis* and the small elapid *Drysdalia coronoides* were detected by two methods (substrate and active searches); however, of the 58 *A. maccoyi* observations, 55 (95%) were from substrate searches, and only three were detected in active searches (Table 2). Had we not used substrate searches in our study, observations of these hard‐to‐detect reptiles would have been markedly reduced and altered our modeled results for reptile assemblage. Our findings demonstrate how species’ ecological traits may impact detection (Driscoll, Smith, Blight, & Maindonald, 2012) and validate the importance of using a variety of survey methods.

We recorded 59% of reptile species that have previously been recorded in our greater study area. Our study was limited to forests and woodlands above 738 m and away from water bodies; therefore, we did not expect to record grass‐ and bog‐land species or species occurring only at lower elevations of the national park. Additionally, we did not record *Notechis scutatus*,* Intelligama lesueurii*, or *Liopholis montana* in any of our surveys; however, we did observe an individual from each of these species in our study area but outside our sites. These species generally require specific habitat features, such as wetlands, water bodies, or large rock crevices at high elevations, respectively (Cogger, 2014; Wilson & Swan, 2013), which were not present in our sites.

### 4.4 Management implications

More than 75% of reptiles from all 20 species detected in this study were recorded in the long‐unburned areas (Table 2), which represent one‐third of our sites and <8% of our study area (Supporting Information Figure S1). Forests and woodlands 6–12 years postfire covered the majority of our study area and, along with sites 0.5–2 years postfire, supported a lower richness and abundance of reptiles relative to long‐unburned sites. Areas with longer postfire ages are often prioritized for prescribed burning (Fernandes & Botelho, 2003). Further, prescribed burning typically reduces fire fuel hazard for up to 5 years (Fernandes & Botelho, 2003), so is usually undertaken in relatively frequent intervals. Reducing overall landscape‐scale fuel hazard would require prescribed burning of a frequency and extent (Dixon et al., 2018; Furlaud, Williamson, & Bowman, 2017; Valentine et al., 2012) that would result in fuel ages that are generally detrimental to reptile richness and abundance in our study area.

Historically, fire frequency in the broader region of our study area is estimated to have been at intervals of >50 years (Banks, 1989) and it is surmised that since European settlement fire frequency may have increased as much as sevenfold across much of the Australian Alps (Zylstra, 2006). The relatively small proportion of long‐unburned forest and woodland remaining in our study area may provide key refuges for reptiles. However, given some dissimilarities in reptile composition between the long‐unburned sites and sites 0.5–12 years postfire, it is presumably important to retain forest and woodland stands in all the different fire ages we studied.

Our results suggest that transitioning more of the study area to a longer TSF will almost certainly increase reptile richness and abundance. Thus, we recommend that in order to maximize reptile richness and abundance, future fire management planning should aim to (a) retain the current long‐unburned areas and manage them as an asset to protect, and (b) transition a higher proportion of forests and woodlands to long‐unburned.
