# Supplementary material for: The disproportionate importance of long‐unburned forests and woodlands for reptiles
Source: Ecol Evol. 2018 Oct 17;8(22):10952–63. doi: 10.1002/ece3.4561 (PMC6262929; doi:10.1002/ece3.4561)
Supplement: Supplementary file 1 [file ECE3-8-10952-s001.docx]

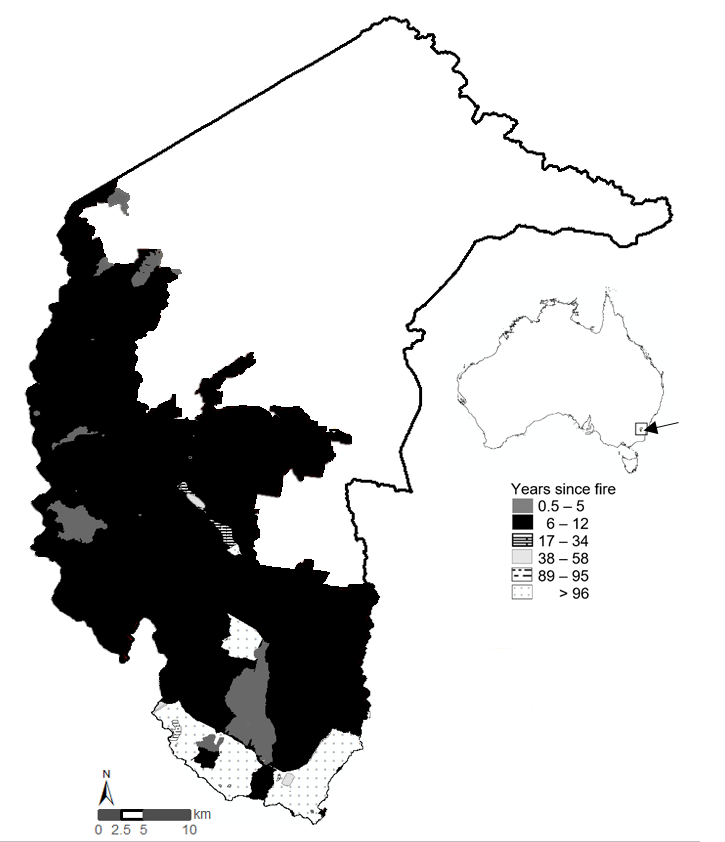


Figure S1 Namadgi National Park (shaded) within the border of the ACT showing time since the last fire (years) within the Park at the time of sampling.

Table S1 Variables measured but excluded from analyses due to correlation with other variables

| Variable | Description |
| --- | --- |
| Litter depth | The average litter depth (mm) taken from five measurements in a one-metre radius (plot) and the average of ten plots per transect (50 m) using a depth gauge made from a 30 cm ruler with a cardboard disk as per Hines et al. (2010). |
| Shrub height | The average height of shrubs and vegetation (> 50 – 300 cm high) within a 2 m radius plot from ten plots per transect (50 m). |
| Number of fires | Total number of fires in mapped fire history (since 1920). |
| Elevation | Elevation of site (m asl) derived from a 20 m resolution digital elevation model developed by John Stein at the Australian National University. |
| Mean warm temperature | Mean annual warmest temperature (degrees Celsius) based on ANUCLIM 1976 to 2005 observations (Xu & Hutchinson, 2011). |
| Mean cold temperature | Mean annual coldest temperature (degrees Celsius) based on ANUCLIM 1976 to 2005 observations (Xu & Hutchinson, 2011). |

Table S2 Coefficient estimates and their standard errors for model 1.1, Table 3.

| Variable | Parameter estimate | SE |
| --- | --- | --- |
| *Reference level 0.5 to 2 years since fire* |  |  |
| Intercept | 1.373 | 0.116 |
| Time since fire 0.5 to 2 years | 0.000 | 0.000 |
| Time since fire 6 to 12 years | -0.201 | 0.149 |
| Time since fire > 96 years | 0.836 | 0.132 |
| *Reference level > 96 years since fire* |  |  |
| Intercept | 2.209 | 0.064 |
| Time since fire > 96 years | 0.000 | 0.000 |
| Time since fire 0.5 to 2 years | -8.365 | 0.132 |
| Time since fire 6 to 12 years | -1.037 | 0.114 |

Table S3 Candidate models fitted to predict reptile richness. Models with ΔAIC_c_ < 2 have the greatest support. Weight (W_i_) is the probability of that model being the best fitting for the data. TSF: time since fire; CWD: coarse woody debris; Veg: forest type

| Model | *df* | Log-likelihood | AICc | ΔAICc | AICc  W_i_ |
| --- | --- | --- | --- | --- | --- |
| 1.1 TSF | 3 | -160.380 | 327.1 | 0.00 | 0.229 |
| 1.2 TSF + rocks + ground cover | 5 | -158.412 | 327.6 | 0.55 | 0.174 |
| 1.3 TSF + ground cover | 4 | -159.674 | 327.9 | 0.80 | 0.153 |
| 1.4 TSF + CWD | 4 | -160.152 | 328.8 | 1.76 | 0.095 |
| 1.5 TSF + litter cover | 4 | -160.320 | 329.2 | 2.10 | 0.080 |
| 1.6 TSF + shrub cover | 4 | -160.363 | 329.3 | 2.18 | 0.077 |
| 1.7 TSF + CWD + rock cover | 5 | -159.382 | 329.6 | 2.49 | 0.066 |
| 1.8 TSF + CWD + ground cover | 5 | -159.429 | 329.7 | 2.59 | 0.063 |
| 1.9 TSF + CWD + shrub cover | 5 | -160.149 | 331.1 | 4.03 | 0.031 |
| 1.10 TSF + CWD + ground cover + shrub cover | 6 | -159.421 | 332.0 | 4.91 | 0.020 |
| 1.11 TSF + CWD + litter cover + ground cover + shrub cover | 7 | -159.277 | 334.1 | 7.02 | 0.007 |
| 1.12 TSF + Veg + Aspect | 8 | -159.570 | 337.1 | 10.07 | 0.001 |
| 1.13 TSF + Veg + Aspect + ground cover | 9 | -158.705 | 337.9 | 10.88 | 0.001 |
| 1.14 TSF + Veg + Aspect + CWD | 9 | -159.282 | 339.1 | 12.03 | 0.001 |
| 1.15 TSF + Veg + Aspect + litter cover | 9 | -159.373 | 339.3 | 12.21 | 0.001 |
| 1.16 TSF + Veg + Aspect + shrub cover | 9 | -159.510 | 339.6 | 12.49 | 0.000 |
| 1.17 TSF + Veg + Aspect + CWD + shrub cover | 10 | -159.163 | 341.5 | 14.40 | 0.000 |
| 1.19 TSF + Veg + Aspect + CWD + rock cover + shrub cover + ground cover + litter cover | 13 | -155.309 | 342.1 | 14.98 | 0.000 |
| 1.18 TSF + Veg + Aspect + CWD + shrub cover + ground cover | 11 | -158.299 | 342.4 | 15.35 | 0.000 |
| 1.20 Veg + Aspect + CWD | 7 | -180.226 | 376.0 | 48.91 | 0.000 |
| 1.21 Veg + Aspect + shrub cover | 7 | -184.574 | 384.7 | 57.61 | 0.000 |
| 1.22 Veg + Aspect + ground cover | 7 | -192.389 | 400.3 | 73.24 | 0.000 |
| 1.23 Veg + Aspect + litter cover | 7 | -196.856 | 409.2 | 82.18 | 0.000 |

Table S4 Coefficient estimates and their standard errors for model 2.1, Table 3.

| Variable | Parameter estimate | SE |
| --- | --- | --- |
| *Reference level 0.5 to 2 years since fire* |  |  |
| Intercept | -0.828 | 0.937 |
| Time since fire 0.5 to 2 years | 0.000 | 0.000 |
| Time since fire 6 to 12 years | -0.094 | 0.185 |
| Time since fire > 96 years | 1.666 | 0.222 |
| *Reference level > 96 years since fire* |  |  |
| Intercept | 0.838 | 1.055 |
| Time since fire > 96 years | 0.000 | 0.000 |
| Time since fire 0.5 to 2 years | -1.666 | 0.222 |
| Time since fire 6 to 12 years | -1.760 | 0.187 |
| *Reference level (year) 2015* |  |  |
| Year 2015 | 0.000 | 0.000 |
| Year 2016 | 0.243 | 0.084 |
|  |  |  |
| Coarse woody debris volume | 0.202 | 0.090 |
| Ground cover proportion | 0.783 | 0.366 |

Table S5 Candidate models fitted to predict reptile abundance. Models with ΔAIC_c_ < 2 have the greatest support. Weight (W_i_) is the probability of that model being the best fitting for the data. Every model included the fixed effect of year and the random effect of site. TSF: time since fire; CWD: coarse woody debris; Veg: forest type

| Model | *df* | Log-likelihood | AICc | ΔAICc | AICc  Weight |
| --- | --- | --- | --- | --- | --- |
| 3.1 TSF + CWD + shrub cover + ground cover + year | 8 | -544.618 | 1106.2 | 0.00 | 0.301 |
| 3.3 TSF + CWD + shrub cover + ground cover + year | 9 | -543.990 | 1107.2 | 0.99 | 0.184 |
| 3.2 TSF + CWD + litter cover + ground cover + shrub cover + year | 10 | -543.192 | 1107.8 | 1.66 | 0.131 |
| 3.4 TSF + CWD + year | 7 | -546.872 | 1108.5 | 2.29 | 0.096 |
| 3.6 TSF + ground cover + year | 7 | -547.016 | 1108.8 | 2.58 | 0.083 |
| 3.5 TSF + CWD + shrub cover + year | 8 | -546.181 | 1109.3 | 3.12 | 0.063 |
| 2.8 TSF + year | 6 | -548.977 | 1110.5 | 4.32 | 0.035 |
| 2.9 TSF + shrub cover + year | 7 | -547.908 | 1110.5 | 4.37 | 0.034 |
| 2.7 TSF + Veg + Aspect + CWD + ground cover + year | 13 | -541.593 | 1111.6 | 5.47 | 0.020 |
| 2.12 TSF + litter cover + year | 7 | -548.731 | 1112.2 | 6.01 | 0.015 |
| 2.13 TSF + Veg + Aspect + ground cover + year | 12 | -543.335 | 1112.8 | 6.59 | 0.011 |
| 2.11 TSF + Veg + Aspect + CWD + ground cover + shrub cover + year | 14 | -541.084 | 1113.0 | 6.85 | 0.010 |
| 2.10 TSF + Veg + Aspect + CWD + litter cover + ground cover + shrub cover + year | 15 | -540.256 | 1113.8 | 7.62 | 0.007 |
| 2.14 TSF + Veg + Aspect + CWD + year | 12 | -544.564 | 1115.2 | 9.04 | 0.003 |
| 2.15 TSF + Veg + Aspect + CWD + shrub cover + year | 13 | -543.815 | 1116.1 | 9.91 | 0.002 |
| 2.16 TSF + Veg + Aspect + shrub cover + year | 12 | -545.014 | 1116.1 | 9.94 | 0.002 |
| 2.17 TSF + Veg + Aspect + year | 11 | -546.187 | 1116.1 | 9.96 | 0.002 |
| 2.18 TSF + Veg + Aspect + litter cover + year | 12 | -545.953 | 1118.0 | 11.82 | 0.001 |
| 2.19 Veg + Aspect + CWD + year | 10 | -576.027 | 1173.5 | 67.33 | 0.000 |
| 2.20 Veg + Aspect + shrub cover + year | 10 | -576.687 | 1174.8 | 68.65 | 0.000 |
| 2.21 Veg + Aspect + ground cover + year | 10 | -585.439 | 1192.3 | 86.16 | 0.000 |
| 2.22 Veg + Aspect + year | 9 | -589.803 | 1198.8 | 92.61 | 0.000 |

Table S6 SIMPER analysis of reptile dissimilarity showing the variance between fire age categories 0.5 to 2 years and 6 to 12 years.

| Species | Average abundance | | Average dissimilarity | % contribution | Cumulative contribution (%) |
| --- | --- | --- | --- | --- | --- |
|  | 0.5 to 2 | 6 to 12 |  |  |  |
| *Lampropholis guichenoti* | 5.00 | 5.24 | 0.187 | 30.23 | 30.23 |
| *Pseudemoia entrecasteauxii* | 3.58 | 5.48 | 0.182 | 29.51 | 59.74 |
| *Eulamprus* spp. | 1.42 | 0.76 | 0.067 | 10.93 | 70.67 |
| *Anepischetosia maccoyi* | 0.47 | 0.58 | 0.033 | 5.38 | 76.05 |
| *Tiliqua nigrolutea* | 0.32 | 0.39 | 0.025 | 4.09 | 80.14 |
| *Pseudemoia spenceri* | 0.68 | 0.24 | 0.242 | 3.94 | 84.08 |
| *Acritoscincus platynotum* | 0.26 | 0.15 | 0.015 | 2.48 | 86.56 |
| *Acritoscincus duperreyi* | 0.26 | 0.15 | 0.014 | 2.33 | 88.89 |
| *Liopholis whitii* | 0.21 | 0.12 | 0.011 | 1.83 | 90.72 |
| *Amphibolurus muricatus* | 0.00 | 0.21 | 0.010 | 1.65 | 92.37 |
| *Pseudonaja textilis* | 0.21 | 0.30 | 0.010 | 1.63 | 94.00 |
| *Drysdalia coronoides* | 0.15 | 0.09 | 0.009 | 1.42 | 95.42 |
| *Hemiergis talbingoensis talbingoensis* | 0.16 | 0.03 | 0.008 | 1.30 | 96.72 |
| *Egernia saxatilis intermedia* | 0.05 | 0.12 | 0.006 | 1.02 | 97.74 |
| *Rankinia diemensis* | 0.05 | 0.09 | 0.006 | 0.90 | 98.64 |
| *Austrelaps ramsayi* | 0.05 | 0.06 | 0.003 | 0.54 | 99.18 |
| *Varanus rosenbergi* | 0.00 | 0.06 | 0.003 | 0.54 | 99.72 |
| *Lampropholis delicata* | 0.00 | 0.03 | 0.001 | 0.14 | 99.86 |
| *Pseudechis porphyriacus* | 0.00 | 0.03 | 0.001 | 0.14 | 100.00 |
| *Egernia cunninghami* | 0.00 | 0.00 | 0.000 | 0.00 | 100.00 |

Table S7 SIMPER analysis of reptile dissimilarity showing the variance between fire age categories 0.5 to 2 years and >96 years.

| Species | Average abundance | | Average dissimilarity | % contribution | Cumulative contribution (%) |
| --- | --- | --- | --- | --- | --- |
|  | 0.5 to 2 | > 96 |  |  |  |
| *Pseudemoia entrecasteauxii* | 3.57 | 49.37 | 0.392 | 50.06 | 50.06 |
| *Lampropholis guichenoti* | 5.00 | 18.70 | 0.170 | 21.70 | 71.76 |
| *Pseudemoia spenceri* | 0.68 | 12.59 | 0.089 | 11.42 | 83.18 |
| *Lampropholis delicata* | 0.00 | 2.04 | 0.020 | 2.54 | 85.72 |
| *Eulamprus* spp. | 1.42 | 0.96 | 0.018 | 2.13 | 88.03 |
| *Liopholis whitii* | 0.21 | 1.30 | 0.018 | 2.29 | 90.32 |
| *Anepischetosia maccoyi* | 0.47 | 1.11 | 0.012 | 1.64 | 91.96 |
| *Amphibolurus muricatus* | 0.00 | 0.63 | 0.010 | 1.37 | 93.33 |
| *Acritoscincus duperreyi* | 0.26 | 0.81 | 0.009 | 1.20 | 94.53 |
| *Acritoscincus platynotum* | 0.26 | 0.74 | 0.009 | 1.10 | 95.63 |
| *Tiliqua nigrolutea* | 0.32 | 0.44 | 0.007 | 0.88 | 96.51 |
| *Drysdalia coronoides* | 0.16 | 0.44 | 0.006 | 0.79 | 97.30 |
| *Hemiergis talbingoensis talbingoensis* | 0.16 | 0.37 | 0.005 | 0.68 | 97.98 |
| *Pseudonaja textilis* | 0.21 | 0.19 | 0.004 | 0.54 | 98.52 |
| *Egernia cunninghami* | 0.00 | 0.30 | 0.003 | 0.45 | 98.97 |
| *Egernia saxatilis intermedia* | 0.05 | 0.30 | 0.003 | 0.42 | 99.39 |
| *Austrelaps ramsayi* | 0.05 | 0.19 | 0.002 | 0.30 | 99.66 |
| *Rankinia diemensis* | 0.05 | 0.37 | 0.001 | 0.15 | 99.81 |
| *Varanus rosenbergi* | 0.00 | 0.11 | 0.001 | 0.13 | 99.94 |
| *Pseudechis porphyriacus* | 0.00 | 0.04 | 0.000 | 0.06 | 100.00 |

Table S8 SIMPER analysis of reptile dissimilarity showing the variance between fire age categories 6 to 12 years and > 96 years since fire.

| Species | Average abundance | | Average dissimilarity | % contribution | Cumulative contribution (%) |
| --- | --- | --- | --- | --- | --- |
|  | 6 to 12 | > 96 |  |  |  |
| *Pseudemoia entrecasteauxii* | 5.48 | 49.37 | 0.374 | 49.61 | 49.61 |
| *Lampropholis guichenoti* | 5.24 | 18.70 | 0.167 | 22.20 | 71.81 |
| *Pseudemoia spenceri* | 0.24 | 12.59 | 0.088 | 11.70 | 83.51 |
| *Lampropholis delicata* | 0.03 | 2.04 | 0.020 | 2.59 | 86.10 |
| *Liopholis whitii* | 0.12 | 1.30 | 0.017 | 2.25 | 88.35 |
| *Eulamprus* spp. | 0.76 | 0.96 | 0.014 | 1.81 | 90.16 |
| *Amphibolurus muricatus* | 0.21 | 0.63 | 0.011 | 1.51 | 91.67 |
| *Anepischetosia maccoyi* | 0.58 | 1.11 | 0.011 | 1.47 | 93.14 |
| *Acritoscincus duperreyi* | 0.15 | 0.81 | 0.009 | 1.21 | 94.35 |
| *Acritoscincus platynotum* | 0.15 | 0.74 | 0.008 | 1.09 | 95.44 |
| *Tiliqua nigrolutea* | 0.39 | 0.44 | 0.008 | 1.08 | 96.52 |
| *Drysdalia coronoides* | 0.09 | 0.44 | 0.006 | 0.81 | 97.33 |
| *Hemiergis talbingoensis talbingoensis* | 0.03 | 0.37 | 0.005 | 0.62 | 97.95 |
| *Egernia saxatilis intermedia* | 0.12 | 0.30 | 0.003 | 0.51 | 98.46 |
| *Egernia cunninghami* | 0.00 | 0.30 | 0.003 | 0.46 | 98.92 |
| *Austrelaps ramsayi* | 0.06 | 0.19 | 0.002 | 0.28 | 99.20 |
| *Pseudonaja textilis* | 0.06 | 0.19 | 0.002 | 0.20 | 99.48 |
| *Varanus rosenbergi* | 0.06 | 0.11 | 0.002 | 0.22 | 99.70 |
| *Rankinia diemensis* | 0.09 | 0.37 | 0.001 | 0.20 | 99.90 |
| *Pseudechis porphyriacus* | 0.03 | 0.04 | 0.000 | 0.10 | 100.00 |

Table S9 Species detected by the four survey methods

| Survey method | *n* species | Species names |
| --- | --- | --- |
| Active + visual + camera + substrate | 7 | *Lampropholis delicata, L. guichenoti, Acritoscincus duperreyi, A. platynotum, Eulamprus* spp*., Pseudemoia entrecasteauxii, Liopholis whitii* |
| Active + visual + camera | 3 | *Amphibolurus muricatus, Egernia cunninghami, Pseudonaja textilis,* |
| Active + visual + substrate | 1 | *Austrelaps ramsayi* |
| Active + visual | 2 | *Egernia saxatilis intermedia, Pseudemoia spenceri* |
| Active + substrate | 2 | *Hemiergis talbingoensis talbingoensis, Anepischetosia maccoyi* |
| Visual + camera | 3 | *Rankinia diemensis, Tiliqua nigrolutea, Varanus rosenbergi* |
| Visual + substrate | 1 | *Drysdalia coronoides* |
| Active | 1 | *Pseudechis prophyriacus* |


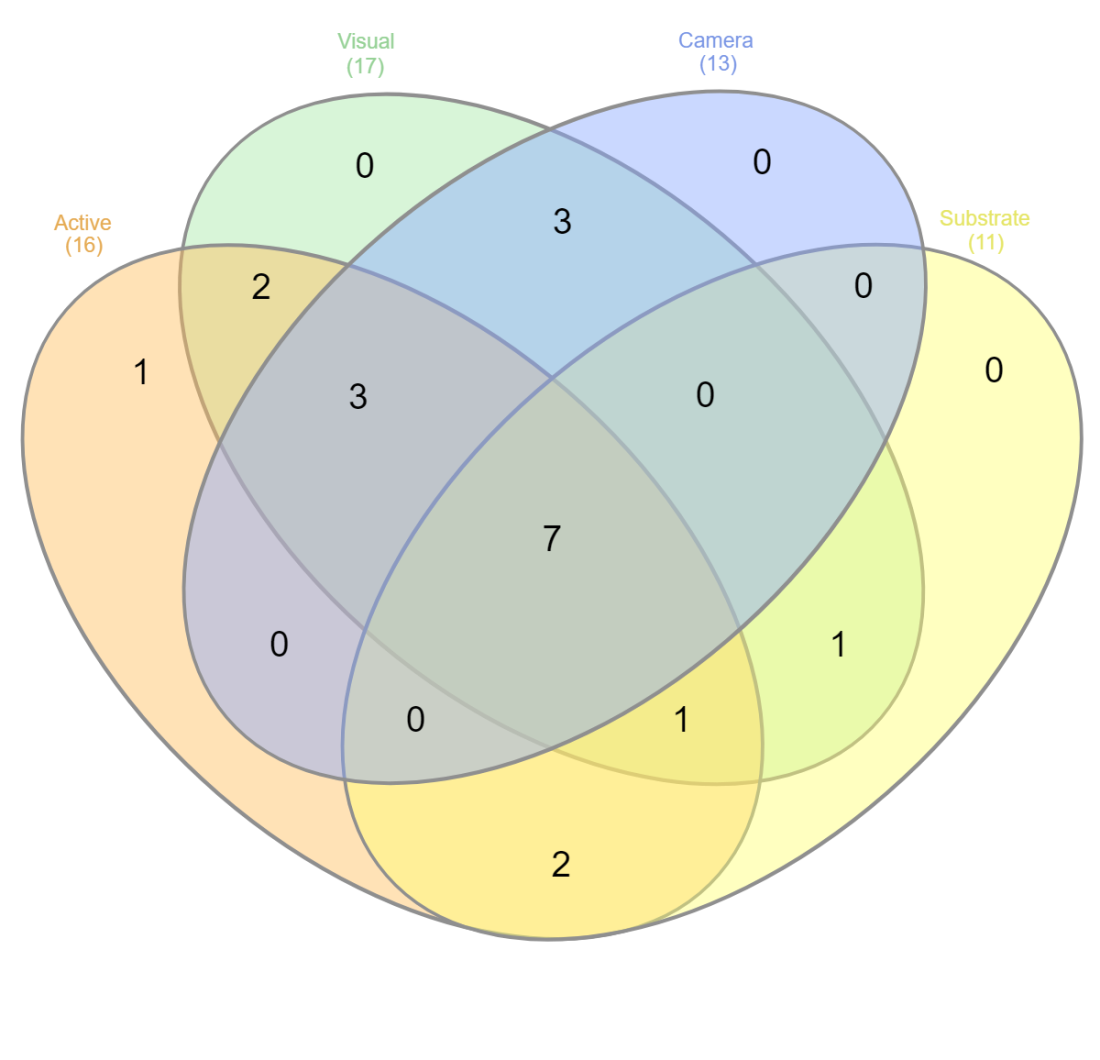


Figure S2 Number of species detected by each survey method and overlap between methods. Venn diagram created using Heberle et al. (2015).

References

Heberle, H., Meirelles, G., da Silva, F., Telles, G. & Minghim, R. (2015) InteractiVenn: a web-based tool for the analysis of sets through Venn diagrams. *BMC Bioinformatics,* **16**.

Hines, F., Tolhurst, K.G., Wilson, A.A. & McCarthy, G.J. (2010) *Overall fuel hazard assessment guide: fire and adaptive management, report number 82,* 4 edn. Victorian Government Department of Sustainability and Environment, Melbourne, VIC, Australia.

Xu, T. & Hutchinson, M. (2011) ANUCLIM Version 6.1. The Australian National University, Canberra, ACT.
